# Clinical and molecular investigation of a canine distemper outbreak and vector-borne infections in a group of rescue dogs imported from Hungary to Switzerland

**DOI:** 10.1186/s12917-015-0471-0

**Published:** 2015-07-16

**Authors:** Barbara Willi, Andrea M. Spiri, Marina L. Meli, Felix Grimm, Laura Beatrice, Barbara Riond, Tim Bley, Rolf Jordi, Matthias Dennler, Regina Hofmann-Lehmann

**Affiliations:** Clinical Laboratory, Vetsuisse Faculty, University of Zurich, Zurich, Switzerland; Center for Clinical Studies, Vetsuisse Faculty, University of Zurich, Zurich, Switzerland; Clinic for Small Animal Internal Medicine, Vetsuisse Faculty, University of Zurich, Zurich, Switzerland; Institute of Parasitology, Vetsuisse Faculty, University of Zurich, Zurich, Switzerland; Clinic of Diagnostic Imaging, Vetsuisse Faculty, University of Zurich, Zurich, Switzerland; Kleintierpraxis Dres. med. vet. Rohner & Bley, Niederglatt, Switzerland; Kleintierpraxis Dr. med. vet. Rolf Jordi, Gümligen, Switzerland

**Keywords:** Canine distemper virus, Outbreak, Domestic dog, Import, Vector-borne infections, Phylogenetic analysis, Vaccination, Arctic-like lineage

## Abstract

**Background:**

Canine distemper virus (CDV) is a major pathogen of dogs and wild carnivores worldwide. In Switzerland, distemper in domestic dogs is rarely reported. In recent years, the import of dogs from Eastern Europe to Switzerland has steadily increased. In the present study, we describe a distemper outbreak in 15 rescue dogs that were imported from Hungary to Switzerland by an animal welfare organisation. The data on vaccination and medical history were recorded (14 dogs), and the samples were collected to investigate CDV and vector-borne infections (13 dogs) and canine parvovirus infection (12 dogs). The dogs were monitored for six months.

**Results:**

One dog was euthanised directly after import. Thirteen dogs showed clinical signs after arrival, i.e., diarrhoea (57 %), coughing (43 %) and nasal and/or ocular discharge (21 %); radiographic findings that were compatible with bronchopneumonia were present in four dogs. CDV infection was diagnosed in 11 dogs (85 %); 10 dogs (91 %) tested PCR-positive in conjunctival swabs. Vector-borne infections (*Babesia spp.*, *Leishmania infantum, Dirofilaria immitis*) were found in 4 dogs (31 %). Three dogs were hospitalized, and six dogs received ambulatory therapy for up to two months until recovery. None of the dogs developed neurological disease. CDV shedding was detected for a period of up to four months. Because dogs were put under strict quarantine until CDV shedding ceased, CDV did not spread to any other dogs. The CDV isolates showed 99 % sequence identity in the HA gene among each other and belonged to the Arctic-like lineage of CDV.

**Conclusions:**

The present study highlights the imminent risks of spreading contagious viral and vector-borne infections through the non-selective import of sick dogs and dogs with incomplete vaccination from Eastern Europe. CDV shedding was detected for several months after the cessation of clinical signs, which emphasised the roles of asymptomatic carriers in CDV epidemiology. A long-term follow-up using sensitive PCR and strict quarantine measures is of upmost importance in preventing the spread of infection. Dog owners and animal welfare organisations should be educated regarding the importance of complete vaccinations and the impact of dog imports on the spread of viral and vector-borne pathogens.

## Background

Canine distemper virus (CDV) is one of the most important viral pathogens in domestic dogs and causes high morbidity and mortality worldwide, particularly in unvaccinated dogs or dogs with incomplete vaccination [1]. CDV is a small, enveloped RNA virus that belongs to the family *Paramyxoviridae* and the genus *Morbillivirus* [2]. CDV has a wide natural host range that includes a variety of terrestrial carnivores [2]. Dogs are thought to be the major reservoir host for CDV [3, 4]. Infection occurs by direct contact with oronasal secretions of infected animals [5]; indirect transmission plays only a minor role in CDV epidemics because the virus is quickly inactivated in the environment [6].

The course of the CDV infection is strongly dependent on the immune response in infected animals [7]. In this context, vaccination is critically important. Dogs that develop an adequate immune response can clear the virus from most tissues, whereas in dogs that show an intermediate immune response, CDV infects the epithelial tissues and induces clinical signs. In dogs that have a weak immune response, CDV disseminates to various tissues, and the clinical signs are usually severe with the persistence of the virus until death [8]. Invasion of the central nervous system occurs when viraemia is sufficiently high [9, 10]. More than 50 % of all CDV infections are perceived to be subclinical [11]. In clinically affected dogs, the disease usually starts with fever and a serous-to-mucopurulent conjunctivitis, followed by a dry to productive cough, depression, anorexia, vomiting and diarrhoea [1]. Neurological signs usually develop within one to three weeks after recovery from systemic illness, but can occur weeks to months later [12].

Since the introduction of highly protective CDV modified live virus (MLV) vaccines more than 60 years ago [13], the incidence of CDV infection in completely vaccinated dogs has decreased [8]. However, in regions with a low proportion of vaccinated dogs, in stray dogs and in shelter environments, the incidence of CDV epidemics is high. In Switzerland, the last CDV epidemic in domestic dogs occurred in 1984–1985; this outbreak was suspected to be attributed to an inadequate vaccination rate in the Swiss dog population at that time [14]. Furthermore, a CDV epidemic associated with high morbidity and mortality commenced in the spring of 2009 in wild carnivores in Switzerland [15]. The latter was perceived to be part of a large transnational outbreak that spread from Eastern to Western Europe. Only one domestic dog was affected in Switzerland during this outbreak [15]. Remarkably, the 2-year-old mixed breed dog died of a CDV-associated neurological disease, although it had received the standard anti-CDV vaccination protocol.

According to the Animal Identity Service (ANIS) in Switzerland, the import of dogs increased by 23 % within one year (2011–2012) [16]. The imported dogs comprised primarily stray dogs that were adopted by animal welfare organizations or pure breed dogs to meet the increasing demand of miniature breeds in Western Europe. In the present study, we report on a distemper outbreak in rescue dogs that had been imported from Hungary to Switzerland. The study provides data on vaccination, medical history, clinical examinations and diagnostic imaging of the dogs and CDV testing, testing for canine parvovirus (CPV) and vector-borne infections. Additionally, the study gives prospectively collected follow-up data on the treatment, clinical course and outcome of the infections and the period of CDV shedding. Finally, a molecular characterization of the CDV isolates was performed.

## Results

### Import and clinical history of the dogs

**Fig. 1:**
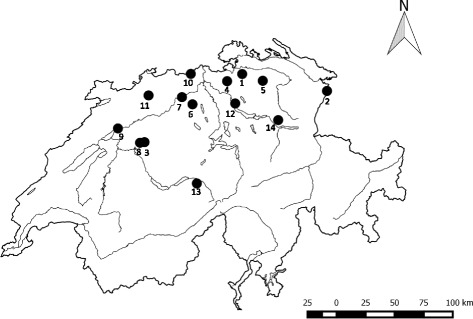
Map of Switzerland showing the geographical distribution of the rescue dogs. Numbers 1 to 14 indicate the location of the 14 dogs (see also Table 1)

A group of 15 rescue dogs that derived from a shelter in Kecskemét, Hungary, was imported to Switzerland in October 2013. One dog was euthanised within several days of import because of clinical deterioration; no data regarding this dog were available. The other 14 dogs comprised nine female (5 spayed) and five male (4 castrated) mixed breed dogs, aged 6 months to 8 years old, and weighing 5 kg to 30 kg (Table 1). All of the dogs had received rabies vaccination (Rabisin®, Biokema SA, Crissier, Switzerland) six to 33 weeks before import and deworming (containing Praziquantel, Pyrantel and Fenbendazol, Uniwerm®, PROVET, Beograd, Serbia) seven to nine days before their arrival in Switzerland. Additionally, the dogs had been vaccinated with one shot of a combined MLV vaccine containing CDV, Canine Adenovirus-2, CPV, *Leptospira* spp. and canine Parainfluenzavirus (Biocan® DHPPi & L, Table 1), either seven to eight days (Dogs 1 to 6 and 8 to 14) or one month prior to arrival in Switzerland (Dog 7); Dog 12 had been revaccinated in Switzerland one week prior to sample collection for CDV PCR (Table 1). After arrival on October 22, 2013, the rescue dogs were directly distributed to 14 private households throughout Switzerland (Table 1, Fig. 1). Seven dogs (Dogs 3, 6, 7, 8, 9, 13 and 14) were placed in multidog households. After arrival, the new owners observed clinical signs in 13 of the 14 dogs (Table 1), i.e., diarrhoea (57 %), coughing (43 %), nasal and/or ocular discharge (21 %), vomiting (14 %), gagging (14 %), lameness (14 %), apathy and sneezing (each 7 %). Because of these symptoms, five dogs (Dogs 1, 2, 3, 4 and 8) were presented to private veterinarians within one week of arrival. Three of these dogs (Dogs 1, 2 and 3) were subsequently referred to small animal clinics for additional investigations.

**Table 1:** Signalment, vaccination history, anamnesis, clinical examination and therapy of the 14 rescue dogs

Dog^1^	Town^2^	Gender^3^	Age^4^	Breed	CDV vaccination	Symptoms after arrival (22.10.13)	Date of CE^7^	Findings at CE^7^	Therapy^8^
**Dog 1**	Aesch	fs	2 y	Mixed breed	15.10.13^**5**^	Diarrhoea	28.10.13	Fever, ocular/nasal discharge, coughing, tachypnoea	Infusion, amoxicillin/clavulanic acid (iv, po), inhalation
**Dog 2**	Widnau	f	8 m	Mixed breed	15.10.13^**5**^	Diarrhoea, coughing, ocular/nasal discharge	5.11.13	Fever, ocular/nasal discharge, coughing, dehydration, increased inspiratory lung sounds, fluid-filled bowel loops	Amoxicillin/clavulanic acid (po), tobramycin eye drops
**Dog 3**	Hettiswil	fs	2 y	Mixed breed	15.10.13^**5**^	Apathy, coughing, nasal discharge	22.10.13	Fever, paleness, purulent-bloody nasal discharge, increased inspiratory lung sounds, diarrhoea	Infusion, amoxicillin/clavulanic acid (iv, po), marbofloxacine (iv, po), imidocarb diproprionate (sc)
**Dog 4**	Niederglatt	mc	3 y	Mixed breed	15.10.13^**5**^	Diarrhoea, coughing, gagging	22.10.13	Fever, purulent ocular discharge, coughing, increased inspiratory lung sounds, conjunctivitis	Infusion, amoxicillin/clavulanic acid (iv, po), allopurinol, inhalation
**Dog 5**	Aadorf	m	6 m	Mixed breed	15.10.13^**5**^	None	5.11.13	Fluid-filled bowel loops	Amoxicillin/clavulanic acid (po), metronidazole (po), diet, deworming
**Dog 6**	Unterkulm	f	6 m	Mixed breed	15.10.13^**5**^	Diarrhoea, sneezing	5.11.13	Conjunctivitis, fluid-filled bowel loops	Amoxicillin/clavulanic acid (po)
**Dog 7**	Niedergösgen	f	7 m	Mixed breed	18.09.13^**5**^	Diarrhoea, coughing, gagging	5.11.13	Coughing, increased inspiratory lung sounds	Amoxicillin/clavulanic acid (po)
**Dog 8**	Urtenen-Schönbühl	f	8 y	Mixed breed	14.10.13^**5**^	Coughing, lameness	23.10.13	Paleness, cachexia, otitis externa	Doxycycline (po), ear drops (Aurizon®), imidocarb diproprionate (sc)
**Dog 9**	Biel	fs	1y	Mixed breed	15.10.13^**5**^	Vomiting	6.11.13	Mucous vaginal discharge	Unknown
**Dog 10**	Rheinsulz	mc	3 y	Mixed breed	14.10.13^**5**^	Diarrhoea, nasal discharge	7.11.13	Conjunctivitis, purulent/mucous ocular/nasal discharge	Doxycycline (po)
**Dog 11**	Zullwil	fs	2 y	Mixed breed	15.10.13^**5**^	Lameness	6.11.13	Lameness	None
Dog 12	Küsnacht	fs	2y	Mixed breed	14.10.13^**5**^, 5.11.13^6^	Vomiting	24.10.13	Normal	None
Dog 13	Meiringen	mc	3 y	Mixed breed	15.10.13^**5**^	Diarrhoea	12.11.13	Normal	None
Dog 14	Rufi	mc	2 y	Mixed breed	15.10.13^**5**^	Diarrhoea, coughing	-	-	Antibiotic injection^9^ (sc)

^1^CDV positive dogs (at first presentation) are shown in bold, no clinical examination could be performed in Dog 14 due to aggressive behaviour; ^2^see also Fig. 1; ^3^ *m* male intact, *mc* male castrated, *f* female intact, *fs* female spayed; ^**4**^ *y* year, *m* month(s); ^**5**^vaccination with Biocan DHPPi & L; ^**6**^vaccination with Nobivac DHPPi; ^**7**^ *CE* clinical examination; ^**8**^ *po* per os, *iv* intravenous, *sc* subcutaneously; ^9^ the antibiotic compound used in Dog 14 was unknown

### Clinical examination findings

Clinical examinations were performed on Dogs 1 to 13 (Table 1); Dog 14 could not be examined because of aggressive behaviour. The clinical examinations revealed mucous, purulent or bloody ocular and/or nasal discharge (39 %), fever (31 %), coughing (31 %), inspiratory increased lung sounds (31 %), conjunctivitis (23 %), fluid-filled bowel loops (23 %), paleness (15 %), and lameness, tachypnoea, diarrhoea, dehydration, cachexia, otitis externa and vaginal discharge in single dogs (Table 1). The clinical examinations were unremarkable in Dogs 12 and 13.

### Haematology and blood biochemistry

Haematology and blood biochemistry results were available for 13 dogs (Tables 2 and 3), 11 of which were CDV-PCR positive (Dogs 1 to 11, see below). At initial presentation, anaemia (9/13, 69 %), leucocytosis (8/13, 62 %), eosinophilia (8/11, 73 %), neutrophilia (6/11, 55 %) and monocytosis (5/12, 42 %) were common (Table 2). Dogs 3 and 4 showed severe pancytopenia and moderate bicytopenia, respectively; both were CDV PCR-positive, co-infected with *Babesia spp.* (Dog 3) or positive for anti-*Leishmania infantum* antibodies (Dog 4, see below), and they exhibited fever, increased inspiratory lung sounds, purulent ocular and nasal discharge and radiographic signs that were compatible with bronchopneumonia (Tables 1 to 5). Dog 8 showed slight anaemia and leucopenia (Table 2); this animal was co-infected with CDV and *Babesia spp.* (Tables 4 and 5). Dog 13 showed a pronounced eosinophilia (Table 2); this animal was CDV-PCR negative but *Dirofilaria immitis* positive (Tables 4 and 5). Blood biochemistry results revealed only unspecific changes in the dogs (Table 3).

**Table 2:** Haematology results of 13 rescue dogs at first presentation

Dog^1^	Date	PCV^2^ (Ref^3^) %	Leuc^4^ (Ref^3^) × 10^3^/μl	Plat^5^ (Ref^3^) × 10^3^/μl	Band Neutro^6^ (Ref^3^) × 10^3^/μl	Segm Neutro^7^ (Ref^3^) × 10^3^/μl	Lymph^8^ (Ref^3^) × 10^3^/μl	Mono^9^ (Ref^3^) × 10^3^/μl	Eos^10^ (Ref^3^) × 10^3^/μl	Baso^11^ (Ref^3^) × 10^3^/μl
**Dog 1**	28.10.13	**32** (42–55)	5.6 (4.7–11.3)	131 (130–394)	0.06 (−0.08)	4.11 (2.5–7.4)	**1.07** (1.2–3.4)	0.37 (0.2–0.9)	**0.03** (0.1–1.3)	0 (−0.08)
**Dog 2**	5.11.13	**34** (42–55)	**11.8** (4.7–11.3)	157 (130–394)	0 (−0.08)	**8.7** (2.5–7.4)	**0.7** (1.2–3.4)	0.78 (0.2–0.9)	**1.57** (0.1–1.3)	0.02 (−0.08)
**Dog 3**	23.10.13	**28** (39–57)	**1.03** (6.0–12.0)	**17** (150–400)	**0.33** (−0.3)	**0.25** (3.0–11.5)	**0.28** (1.0–4.8)	0.17 (0.15–1.4)	**0** (0.1–1.3)	0 (−0.04)
**Dog 4**	22.10.13	**36** (42–57)	9.9 (5.7–12.4)	**88** (200–400)						
**Dog 5**	5.11.13	**33** (42–55)	**17.9** (4.7–11.3)	341 (130–394)	0 (−0.08)	**13.1** (2.5–7.4)	1.67 (1.2–3.4)	**1.43** (0.2–0.9)	**1.64** (0.1–1.3)	0.05 (−0.08)
**Dog 6**	5.11.13	**36** (42–55)	**13.2** (4.7–11.3)	131 (130–394)	0 (−0.08)	**9.23** (2.5–7.4)	2.31 (1.2–3.4)	**1.1** (0.2–0.9)	0.51 (0.1–1.3)	0.05 (−0.08)
**Dog 7**	5.11.13	45 (42–55)	**14.3** (4.7–11.3)	**430 (130–394)**	0 (−0.08)	**7.84** (2.5–7.4)	2.03 (1.2–3.4)	**1.36** (0.2–0.9)	**2.89** (0.1–1.3)	**0.13** (−0.08)
**Dog 8**	23.10.13	**33** (37–55)	**5.44** (6.0–17.0)	**619** (150–500)			**0.5** (1.0–4.8)	0.1 (0.1–1.8)		
**Dog 9**	6.11.13	42 (42–55)	**18.1** (4.7–11.3)	146 (130–394)	0 (−0.08)	**12.61** (2.5–7.4)	2.09 (1.2–3.4)	**1.33** (0.2–0.9)	**1.96** (0.1–1.3)	0.06 (−0.08)
**Dog 10**	7.11.13	**31** (42–55)	9.9 (4.7–11.3)	**418 (130–394)**	0 (−0.08)	3.68 (2.5–7.4)	1.77 (1.2–3.4)	**1.19** (0.2–0.9)	**3.21** (0.1–1.3)	0.06 (−0.08)
**Dog 11**	6.11.13	42 (42–55)	**13.4** (4.7–11.3)	231 (130–394)	0 (−0.08)	6.06 (2.5–7.4)	**4.75** (1.2–3.4)	0.85 (0.2–0.9)	**1.62** (0.1–1.3)	**0.13** (−0.08)
Dog 12	24.10.13	46 (38–55)	**19.0** (6.0–12.0)	159 (150–500)		**10.17 (**3.0–10.0)	**7.24** (1.0–4.0)	0.61 (0–1.2)	**0.95 (**0–0.6)	0.04 **(−**0.04)
Dog 13	12.11.13	**38** (42–55)	**11.4** (4.7–11.3)	250 (130–394)	0.06 (0 – 0.08)	**2.23** (2.5–7.4)	2.4 (1.2–3.4)	0.51 (0.2–0.9)	**6.11** (0.1–1.3)	**0.11** (−0.08)
**Total (%)**	**Increased**		**8/13 (62 %)**	**3/13 (23 %)**	**1/10 (10 %)**	**6/11 (55 %)**	**2/12 (17 %)**	**5/12 (42 %)**	**8/11 (73 %)**	**3/11 (27 %)**
	**Decreased**	**9/13 (69%)**	**2/13 (15 %)**	**2/13 (15 %)**		**2/11 (18 %)**	**4/12 (33 %)**		**2/11 (18 %)**	

Values outside the reference range are shown in bold

^1^CDV positive dogs (at first presentation) are shown in bold, no samples could be collected from Dog 14 (not shown) due to aggressive behaviour; ^**2**^ *PCV* packed cell volume; ^3^Reference range; ^**4**^ *Leuc* leucocytes; ^**5**^ *Plat* platelets; ^**6**^ *Band Neutro* banded neutrophils; ^**7**^ *Segm Neutro* segmented neutrophils; ^**8**^ *Lymph* lymphocytes; ^**9**^ *Mono* monocytes; ^**10**^ *Eos* eosinophils; ^**11**^ *Baso* basophils

**Table 3:** Blood biochemistry results of 13 rescue dogs at first presentation

Dog^1^	Date	Bil^2^ (Ref^3^) μmol/l	Urea (Ref^3^) mmol/l	Crea^4^ (Ref^3^) μmol/l	TP^5^ (Ref^3^) g/l	Alb^6^ (Ref^3^) g/l	AP^7^ (Ref^3^) U/l	ALAT^8^ (Ref^3^) U/l	Na^9^ (Ref^3^) mmol/l	K^10^ (Ref^3^) mmol/l	P^11^ (Ref^3^) mmol/l
**Dog 1**	28.10.13	0.6 (−3.5)	4.4 (3.8–5.9)	50 (50–119)	57 (56–71)	30 (29–37)	25 (20–98)	23 (20–93)	152 (152–159)	4.3 (4.3–5.3)	1.13 (1–1.6)
**Dog 2**	5.11.13	0.6 (−3.5)	2.6 (1.6–6.2)	44 (19–79)	58 (56–71)	29 (29–37)	38 (4–252)	28 (20–93)	153 (152–159)	4.6 (4.3–5.3)	1.89 (1.1–2.5)
**Dog 3**	23.10.13	2.5 (−3.9)	3.4 (3.3-10.8)	44 (52–177)	56 (56–73)	**20** (30–41)	**225** (9–132)	**18** (26–126)	**141** (142–154)	4.5 (4.2–5.4)	**0.55** (0.9–1.9)
**Dog 4**	22.10.13	1.7 (−6.8)	3.8 (2.5–8.8)	84 (−133)	58 (54–68)	31 (30–37)	**297** (−240)	63 (0–75)	146 (140–155)	4.7 (3.9–5.4)	1.1 (1.0–1.7)
**Dog 5**	5.11.13	0.2 (−3.5)	2.9 (1.6–6.2)	41 (19–79)	**49** (56–71)	**21** (29–37)	115 (4–252)	24 (20–93)	**150** (152–159)	**5.7** (4.3–5.3)	**2.6** (1.1–2.5)
**Dog 6**	5.11.13	1.1 (−3.5)	4.2 (1.6–6.2)	57 (19–79)	**49** (56–71)	**27** (29–37)	132 (4–252)	29 (20–93)	**151** (152–159)	4.7 (4.3–5.3)	1.75 (1.1–2.5)
**Dog 7**	5.11.13	0.2 (−3.5)	2.3 (1.6–6.2)	45 (19–79)	**54** (56–71)	**27** (29–37)	94 (4–252)	36 (20–93)	152 (152–159)	5.1 (4.3–5.3)	1.93 (1.1–2.5)
**Dog 8**	23.10.13	7.0 (−10)	7.0 (2.5–8.9)	94 (27–124)	73 (54–82)	33 (25–44)	108 (20–150)	46 (10–118)	143 (138–160)	**5.9** (3.7–5.8)	1.84 (0.9–2.1)
**Dog 9**	6.11.13	1.3 (−3.5)	5.6 (3.8–5.9)	84 (50–119)	60 (56–71)	34 (29–37)	35 (20–98)	29 (20–93)	154 (152–159)	4.8 (4.3–5.3)	1.6 (1–1.6)
**Dog 10**	7.11.13	0.3 (−3.5)	**2.9** (3.8–5.9)	58 (50–119)	64 (56–71)	**26** (29–37)	60 (20–98)	26 (20–93)	153 (152–159)	5.0 (4.3–5.3)	**1.8** (1–1.6)
**Dog 11**	6.11.13	1.4 (−3.5)	**6.7** (3.8–5.9)	69 (50–119)	62 (56–71)	35 (29–37)	40 (20–98)	23 (20–93)	152 (152–159)	**5.4** (4.3–5.3)	**1.92** (1–1.6)
Dog 12	24.10.13	2.5 (−5.4)	6.2 (2.8–10.7)	103 (−133)	64 (52–71)	30 (26–37)	**138** (0–121)	79 (−115)	151 (143–152)	4.7 (3.9–5.4)	1.4 (0.9–2)
Dog 13	12.11.13	1.6 (−3.5)	4.3 (3.8–5.9)	101 (50–119)	59 (56–71)	31 (29–37)	34 (20–98)	31 (20–93)	152 (152–159)	4.7 (4.3–5.3)	**1.8** (1–1.6)
**Total (%)**	**Increased**		**1/13 (8 %)**				**3/13 (23 %)**			**3/13 (23 %)**	**4/13 (31 %)**
	**Decreased**		**1/13 (8 %)**		**3/13 (23 %)**	**5/13 (39 %)**		**1/13 (8 %)**	**3/13 (23 %)**		**1/13 (8 %)**

Values outside the reference range are shown in bold

^1^CDV positive dogs (at first presentation) are shown in bold, no samples could be collected from Dog 14 (not shown) due to aggressive behaviour; ^2^ *Bil* bilirubin; ^3^ *Ref* reference range; ^4^ *Crea* creatinine; ^5^ *TP* total protein; ^6^ *Alb* albumin; ^7^ *AP* alkaline phosphatase; ^8^ *ALAT* alanine aminotransferase; ^9^ *Na* sodium; ^10^ *K* potassium; ^11^ *P* phosphorus

**Table 4:** CDV PCR results at first presentation and at different time points thereafter in 13 rescue dogs

Dogs^1^	Date of first investigation	CDV real-time RT-qPCR results^2^															
		At first investigation			After 1 month	After 2 months			After 3 months			After 4 months			After 5 months		
		CS	NS	Blood	CS/NS/OC^6^	CS	NS	OC	CS	NS	OC	CS	NS	OC	CS	NS	OC
**Dog 1**	28.10.13	**pos**		**pos**	**pos**				**pos**	**pos**	neg	**pos**	**pos**	neg	neg	neg	neg
**Dog 2**	5.11.13	**pos**	**pos**	**pos**	**pos**				neg	**pos**	neg	neg	**pos**	neg	neg	neg	neg
**Dog 3**	6.11.13^3^	**pos**	neg	**pos**	**pos**				neg	neg	neg						
**Dog 4**	22.10.13	**pos**															
**Dog 5**	5.11.13	**pos**	**pos**	**pos**	neg												
**Dog 6**	5.11.13	**pos**	**pos**	**pos**	**pos**	**pos**	**pos**	neg	**pos**	neg	neg	neg	neg	neg			
**Dog 7**	5.11.13	neg	**pos**	**pos**	quest^7^	neg	neg	neg									
**Dog 8**	6.11.13^4^	**pos**	**pos**	neg	**pos**	neg	neg	neg									
**Dog 9**	6.11.13	**pos**		neg	**pos**	neg	neg	neg									
**Dog 10**	7.11.13	**pos**	neg	neg	**pos**	**pos**	**pos**	**pos**	**pos**	**pos**	neg						
**Dog 11**	6.11.13	**pos**	neg	neg	neg												
Dog 12	12.11.13^5^	neg	neg														
Dog 13	12.11.13	neg	neg	neg													
**Total positive**		**11/13**			**7/10**	**2/5**			**4/5**			**2/3**			**0/2**		

Positive results are shown in bold

^1^CDV PCR-positive dogs (at first investigation) are shown in bold, no samples could be collected from Dog 14 (not shown) due to aggressive behaviour. ^2^ *CS* conjunctival swab, *NS* nasal swab, *OC* oropharyngeal swab, *pos* positive result, *neg* negative result, *quest* questionable result. ^*3*^Dog 3 had already tested CDV PCR-positive in a CS collected at first presentation (23.10.13) by the private veterinarian. ^4^Samples for CDV PCR were collected for the first time two weeks after first presentation (23.10.13). ^5^Dog 12 had already tested CDV PCR-negative in blood collected by the private veterinarian at first presentation (24.10.13). ^6^Conjunctival, nasal and oropharyngeal swabs were pooled for CDV PCR. ^7^PCR result questionable due to low cGAPDH levels (for details, see Methods section)

**Table 5:** Results for CPV and for vector-borne infections in 13 rescue dogs

Dog^1^	Date of sampling	CPV^2,3^	*Babesia spp.* ^2^	*Ehrlichia canis* ^2^	*Leishmania infantum* ^2^	*Dirofilaria immitis* ^2^
**Dog 1**	28.10.13	neg	neg ^5^	neg ^8^	neg ^10^	neg ^12^
**Dog 2**	05.11.13	neg	neg ^5^	neg ^8^	neg ^10^	neg ^12^
**Dog 3**	25.10.13		**pos** ^**4**^			
	06.11.13	neg	**pos** ^5^	neg ^8^	neg ^10^	neg ^12^
**Dog 4**	22.10.13		neg ^4^	neg ^9^	**pos** ^**11**^	
**Dog 5**	5.11.13	neg	neg ^5^	neg ^8^	neg ^10^	neg ^12^
**Dog 6**	5.11.13	neg	neg ^5^	neg ^8^	neg ^10^	neg ^12^
**Dog 7**	5.11.13	neg	neg ^5^	neg ^8^	neg ^10^	neg ^12^
**Dog 8**	23.10.13		**pos** ^**6**^			
	6.11.13	neg	**pos** ^5^	neg ^8^	interm^10^	neg ^12^
**Dog 9**	6.11.13	neg	neg ^5^	neg ^8^	neg ^10^	neg ^12^
**Dog 10**	7.11.13	neg	neg ^5^	neg ^8^	neg ^10^	neg ^12^
**Dog 11**	6.11.13	neg	neg ^5^	neg ^8^	neg ^10^	neg ^12^
Dog 12	24.10.13		interm^7^	neg ^8^	neg ^10^	neg ^13^
	12.11.13	neg				
Dog 13	12.11.13	neg	neg ^4^	neg ^8^	neg ^10^	**pos** ^12,14^
**Total positive (%)**		**0/12 (0 %)**	**2/13 (15 %)**	**0/13 (0 %)**	**1/13 (8 %)**	**1/13 (8 %)**

Positive results are shown in bold

^1^CDV-positive dogs (at first presentation) are shown in bold; no samples could be collected from Dog 14 (not shown) due to aggressive behaviour; ^2^ *pos* positive, *neg* negative, *interm* intermediate result, ^3^Result of CPV real-time qPCR; ^4^Microscopic evaluation of blood smears for the presence of *Babesia spp.* organisms by the private veterinarian (Dog 3) or a commercial laboratory (Dog 4: Alomed, Radolfzell-Böhringen, Germany); ^5^Commercial immunofluorescence antibody test (IFAT) for detecting anti-*Babesia canis* antibodies (MegaFLUO® BABESIA canis, Megacor Diagnostik GmbH, Hörbranz, Austria) performed by the Institute of Parasitology, University of Zurich; ^6^Babesia-specific PCR assay from blood performed by a commercial laboratory (Labor am Zugersee, Hünenberg, Switzerland); ^7^Enzyme-linked immunosorbent assay (ELISA) for the detection of *B. canis* antibodies performed by a commercial laboratory (IDEXX Diavet AG, Bäch, Switzerland); ^8^Commercial IFAT for detecting immunoglobulin G antibodies against *Ehrlichia canis* (Mega Screen Fluoehrlichia canis, Megacor GmbH) performed by the Clinical Laboratory, University of Zurich; ^9^PCR for detecting *E. canis* from blood performed by a commercial laboratory (Alomed); ^10^Published ELISA for the specific detection of anti-Leishmania IgG antibodies [45] performed by the Institute of Parasitology, University of Zurich, using *Leishmania infantum* promastigote stage antigens and goat anti-dog IgG (γ) antibodies conjugated to alkaline phosphatase (Kirkegaard and Perry Lab, Inc., Maryland, USA); ^11^IFAT for detecting anti-*L. infantum* antibodies performed by a commercial laboratory (Alomed); ^12^commercial *Dirofilaria immitis* antigen detection test (DiroCHECK, Synbiotics, Lyon, France) performed by the Institute of Parasitology, University of Zurich; ^13^ELISA for the detection of *D. immitis* antigen performed by a commercial laboratory (IDEXX Diavet AG); ^14^Knott Test for the detection of microfilaria of *D. immitis* performed by the Institute of Parasitology, University of Zurich, as previously published [46]

### Radiographic examinations and echocardiography

**Fig 2:**
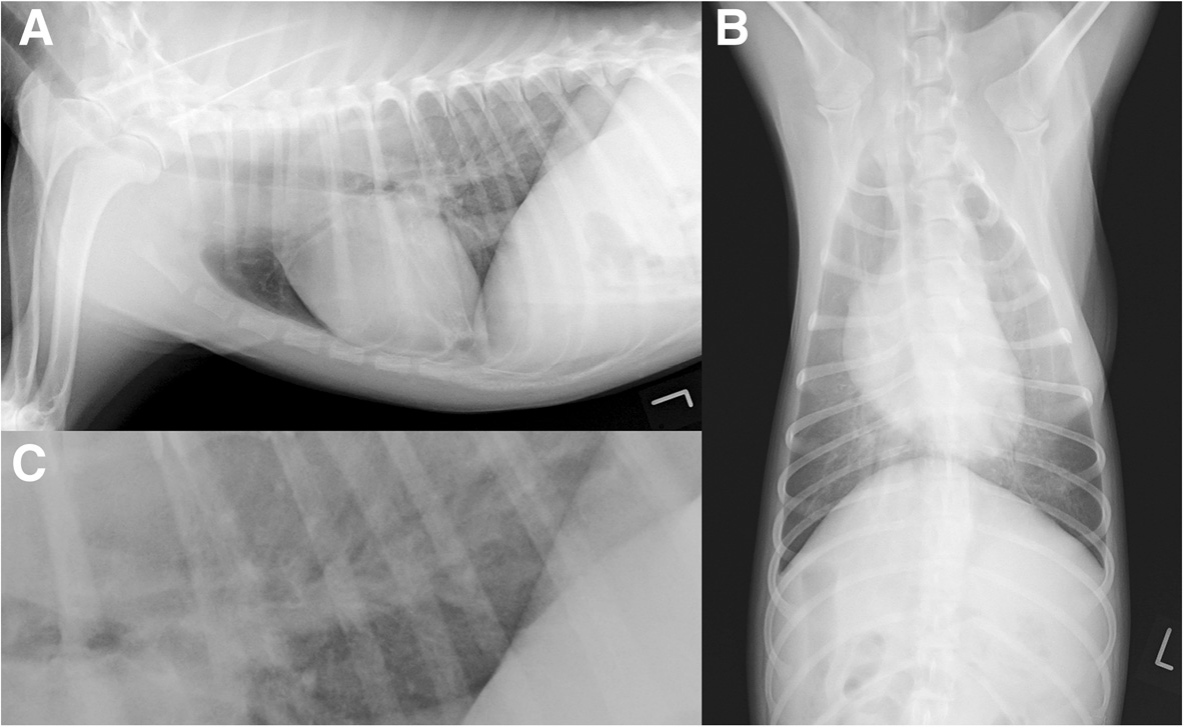
Diagnostic imaging of the thorax of Dog 2. **a** Right to left lateral, (**b**): ventrodorsal, and (**c**): close-up of the lateral projection in the caudodorsal lung field of Dog 2 showing a generalized, moderate, mildly heterogeneous increase in pulmonary opacity, numerous thickened bronchial ring shadows and reduced delineation of the peripheral pulmonary vessels. These generalized moderate broncho-interstitial lung changes are compatible with a moderate bronchopneumonia or bronchitis

The radiographic examinations of the thorax revealed moderate-to-severe interstitial lung changes with variable bronchial thickening in four dogs (Dogs 1 - 4). The changes were generalised and most pronounced in the dorsal (Dog 2), perihilar (Dogs 2 and 4) and caudal lung areas (Dog 3, Fig. 2). The radiographic findings were compatible with bronchopneumonia in all four dogs, and all of the dogs tested CDV PCR-positive. The thoracic radiographs of Dog 13 revealed mild right-sided cardiomegaly and mild generalised bronchointerstitial lung changes; echocardiography showed mild tricuspid and aortic regurgitation but no signs of pulmonary hypertension or right ventricular pressure overload. Dog 13 tested *D. immitis*-positive but was negative for CDV.

### CDV PCR and sequencing results

**Fig. 3:**
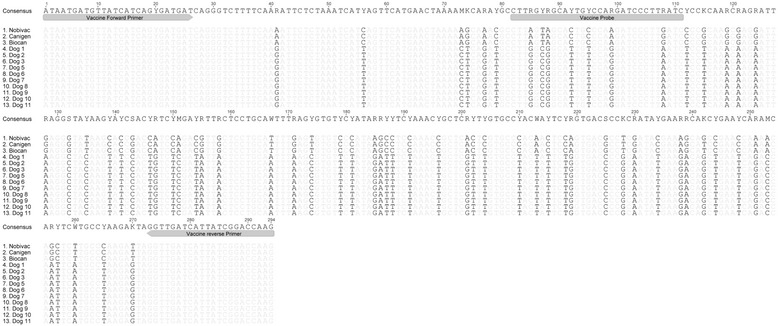
Sequence alignment of CDV vaccine strains and CDV isolates of the rescue dogs. The sequence alignment of the M gene and M-F intergenic region of three CDV vaccine strains (contained in Nobivac® DHHPi, MSD Animal Health; Canigen® SHA2PPi, Virbac; and Biocan® DHPPi & L, Bioveta) and of ten wild-type CDV isolates of the rescue dogs (Dogs 1 to 3 and 5 to 11) is shown. Grey-shaded letters: identical nucleotides between vaccine strains and wild-type isolates. Black letters: nucleotides that differ between vaccine strains and wild-type isolates. Grey bars: schematic drawing depicting the positions of the primers and the probe used in the CDV vaccine specific real-time RT-qPCR assay [17]. Consensus: Consensus sequence of 100 % identical bases matching all of the sequences (most of the ambiguities)

Eleven of the 13 dogs tested CDV PCR-positive during the initial examination (Table 4). The positive PCR results were most commonly obtained from conjunctival swabs (10 of the 11 CDV-positive dogs, Table 4). The vaccine-specific real-time reverse transcription (RT)-quantitative (q)PCR was negative for all ten dogs that were tested, which supports the finding of infection with a wild-type CDV strain. All three vaccines that were tested (Biocan® DHPPi & L, Bioveta, Ivanovice na Hané, Czech Republic; Nobivac® DHHPi, MSD Animal Health, Luzern, Switzerland; Canigen® SHA2PPi, Vibac, Glattbrugg, Switzerland) exhibited a positive PCR result. In gel electrophoresis of the PCR products, a appropriate-sized band was detected for all ten dogs, as were the three vaccines, as expected [17]. The sequence of the amplification product of the Biocan® DHPPi & L vaccine used in the dogs of the present study was clearly distinct from the sequence of the CDV isolates of the ten rescue dogs (Fig. 3) and most closely related to the CDV vaccine strain Onderstepoort (99 % nucleotide identity to AB250738).

**Fig. 4:**
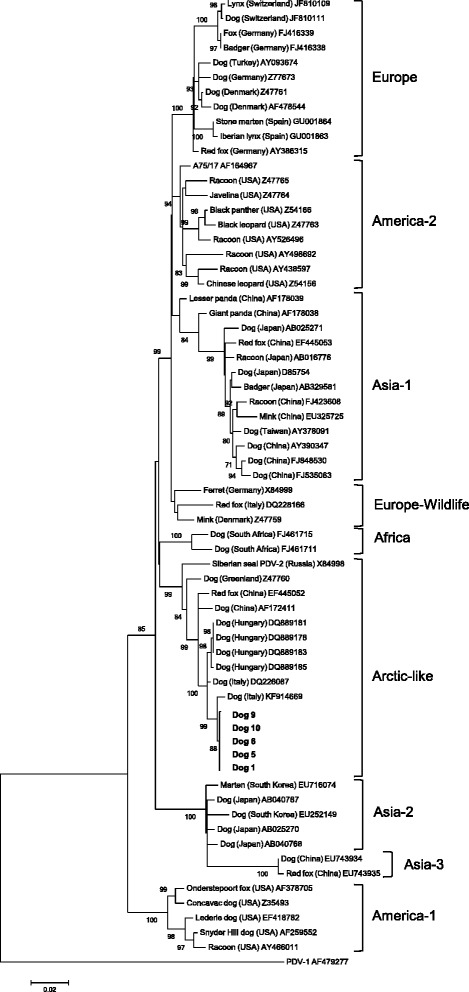
Phylogenetic relationship between selected CDV strains based on the complete haemagglutinin (HA) gene sequence. The CDV isolates analysed in this study appear in bold. Nine CDV lineages are shown: Asia-1, Europe, America-2, Europe-Wildlife, Africa, Arctic-like, Asia-2, Asia-3 and America-1. Phocine distemper virus (PDV-1) was used as the outgroup. GenBank accession numbers, host species and geographical origin are indicated, if known. The numbers at the nodes were generated from 1000 bootstrap resamplings; only values > 70 are shown. The bar represents the mean number of differences per 200 sites. Strain AF178038 (giant panda isolate) is the resultant of a genetic recombination between “Asia-1” and a “Europe-Wildlife” strain [44]

Sequencing of the HA gene of CDV isolates of five of the infected dogs revealed that the dogs were infected with a similar CDV strain (99.9–100 % nucleotide identity among the HA gene of CDV isolates of Dogs 1, 5, 6 and 10); the HA gene of the CDV isolate of Dog 9 differed in only one nucleotide position from the HA gene sequences of the other four CDV isolates. The phylogenetic analysis revealed that the isolates from the five import dogs belonged to the Arctic-like lineage of CDV (Fig. 4). The HA gene sequences of the CDV isolates were most similar to a published HA gene sequence of a CDV strain from a domestic dog from Italy (KF914669, 99 % nucleotide identity, Fig. 4) [18]. They were only distantly related to CDV strains isolated during a CDV epidemic in wild carnivores in Switzerland (JF810109 and JF810111, 92.6 % nucleotide identity, Fig. 4)

### CDV follow-up

Overall, two dogs (Dogs 5 and 11) exhibited CDV PCR-negative results one month after the initial examination (Table 4). Two months after the initial examination, another three dogs were found to be CDV PCR-negative (Dogs 7 to 9); three months after the initial examination, Dog 3 was CDV-negative and the remaining three dogs tested CDV-negative four months (Dog 6) and five months (Dogs 1 and 2) after the initial examination (Table 4).

### Results for CPV and vector-borne infections

All twelve dogs that were tested for CPV at the initial presentation were PCR-negative (Table 5). Vector-borne infections were detected in 4 dogs (31 %, Table 5): infection with *Babesia spp*. was detected in Dogs 3 and 8; infection with *L. infantum* was diagnosed in Dog 4 and infection with *Dirofilaria immitis* was found in Dog 13. Dog 13, which tested positive in *D. immitis* antigen and Knott tests, had received a certificate from a laboratory in Budapest, Hungary, that stated a negative result in the Knott test in August 2013. None of the rescue dogs tested positive for *Ehrlichia canis* (Table 5).

### Therapy, clinical course and outcome

The CDV-infected Dogs 1, 3 and 4 were hospitalized for two to three days and received intravenous infusions of crystalloids, intravenous antibiotic therapy and inhalation (Table 1). Dog 3, which was co-infected with *Babesia spp*., was treated with two imidocarb injections two weeks apart. Dog 4, which was co-infected with *L. infantum* was treated using allopurinol. All three dogs showed rapid clinical improvement with treatment and were discharged with oral antibiotic therapy. Repeated haematological examination in Dog 3 two weeks later revealed that the pancytopenia had resolved and had returned to moderate neutrophilia, eosinophilia and slight monocytosis. Mild anaemia was still present in Dog 3 at that time (data not shown). Repeated haematology in Dog 4 two weeks after the initial presentation showed normal platelets counts and nearly normal PCV values (data not shown). All three dogs were clinically asymptomatic in the 6-month follow-up period.

Dog 2 was ambulatory and was treated with oral antibiotics and antibiotic eye drops (Table 1). The dog showed several relapses with purulent nasal and ocular discharge after antibiotic therapy ceased and was repeatedly treated with antibiotics for two months. Thereafter, there was no relapse, and the dog was clinically asymptomatic in the remaining 4-month follow-up period.

Five CDV PCR-positive dogs (Dogs 5 to 8 and 10) received oral antibiotic therapy (amoxicillin clavulanic acid or doxycycline) for seven to ten days after their arrival in Switzerland. Dog 5 developed watery diarrhoea two weeks after arrival and was additionally treated with metronidazole, deworming and a highly digestible diet. Dog 8, which was co-infected with *Babesia spp.,* received two injections of imidocarb diproprionate two weeks apart and antibiotic ear drops because of otitis externa. At the end of the 6-month follow-up period, all of the CDV PCR-positive dogs had recovered and none had developed neurological signs.

### Confinement of infection

All of the owners of the CDV PCR-positive dogs were instructed by the first author (BW) to quarantine the dogs until they tested CDV PCR-negative. The owners of the CDV-positive dogs in multidog households (Dogs 3, 6, 7, 8 and 9) were instructed to separate the infected dog from the other dogs in the household. However, several dogs (Dogs 6, 7, 8 and 9) had already had contact with adult dogs within the household at the time when the CDV diagnosis was made. All of the contact dogs had been vaccinated against CDV, although several dogs had only received the initial vaccination series as puppies and had received no booster vaccinations (data not shown). Two dogs that were in close contact with Dogs 7 and 9 were tested for the CDV infection with PCR one month and two months after the initial CDV diagnosis in Dogs 7 and 9. One contact dog exhibited a single, very weak CDV-positive result in the conjunctival swab in the first sampling but was negative in all of the swabs collected one month later (data not shown). The other contact dog tested PCR-negative in all of the collected samples (data not shown). In all of the other multidog households, no samples were collected for CDV PCR, but no clinical signs of the disease were noted in the 6-month follow-up period.

## Discussion

The present study describes a distemper outbreak in rescue dogs in Switzerland that had been imported by an animal welfare organization. One dog had to be euthanized directly after import for humane reasons, whereas nine dogs required therapy for up to two months; three of these dogs were hospitalized at veterinary clinics. The imported animals shed CDV for up to four months, which necessitated long-term quarantine measures. Moreover, four of the dogs were infected with vector-borne pathogens.

The present study underscores the risk of introducing contagious or vector-borne pathogens to central European countries by importing rescue dogs with incomplete vaccination. Based on the data of the ANIS in Switzerland, 43.9 % of the newly registered dogs in 2012 were imported [16]. The imported dogs primarily comprised small and miniature purebred dogs to meet the increasing demand for these types of breeds in Switzerland, and mixed breed dogs that are rescued by animal welfare organisations [16]. The dogs of both groups are at risk for carrying infectious diseases because of the inadequate vaccination policies and quarantine measures in many breeding kennels and animal shelters in Eastern Europe. In the animal shelter in Hungary where the dogs originate, no quarantine measures or reliable vaccination policies had been implemented. The dogs had only received a single CDV vaccination either one week or one month before importation to Switzerland. At this time point, several dogs had likely been infected with CDV. After the outbreak, the rescue organisation was instructed to introduce vaccination policies and quarantine measures within the animal shelter.

In Switzerland, the vaccination rate in dogs is insufficiently high to provide population immunity against the CDV infection. The proportion of vaccinated dogs in a population must be > 70 % to provide protection for the dog population, in contrast to protection of only a single vaccinated dog [19]. A vaccination rate of 60–70 % has been estimated for Swiss dogs based on the numbers of sold vaccine doses in 2009 [20]. Mandatory courses for new dog owners have been introduced in Switzerland in which freshly adopted dogs come in close contact with each other. Together with the decreasing vaccination rate and the increasing number of imported dogs, this situation has clearly increased the risk for canine distemper outbreaks. Dog owners and animal welfare organisations should be informed concerning the critical importance of complete vaccination schedules in domestic dogs. In the present outbreak, the infection could be prevented from spreading to other dogs by extensive follow-up examinations of the dogs and a thorough education of the dog owners concerning the critical importance of strict quarantine measures. Luckily, no CDV-positive dogs of this study had already had contact with young unvaccinated dogs at the time of diagnosis. The CDV vaccines are known to induce a strong and long-lasting immunity when no interference with maternally derived antibodies occurs [21, 22].

CDV was reported to be shed by infected animals for up to three months [11, 23]. We demonstrated CDV shedding in some dogs in the present study even for up to four months. Shedding was detected for several months after the cessation of clinical disease. Our results underscore the role of asymptomatic carriers in CDV epidemiology and the importance of a long-term follow-up of CDV-positive dogs for preventing the spread of infection. Several studies have reported the excretion of vaccine strains after CDV vaccination [11, 24]. This was not observed in the present study: the dogs that tested CDV PCR-positive were shown to be infected with wild-type CDV, although the majority of the dogs had received MLV CDV vaccination within one to seven weeks before PCR testing. Consistent with the published data [25], PCR from conjunctival swabs was found to be most reliable for detecting CDV in acutely infected animals and during follow-up examinations. However, in one animal, only the nasal but not the conjunctival swab tested positive for CDV.

Nine of the dogs in the present study were hospitalized or required ambulatory therapy for up to two months. Another animal was euthanised directly after import because of clinical deterioration. In all of the affected dogs, the respiratory and gastrointestinal symptoms predominated. In four dogs, signs of bronchopneumonia were evident. Remarkably, none of the dogs developed a neurological disease. Whether the latter was due to the intrinsic properties of the CDV strain or to the age and immune status of the dogs is unknown. The sequence analyses of the CDV strains detected in the rescue dogs indicated that all of the dogs were infected with the same wild-type CDV strain, which belongs to the Arctic-like lineage of CDV [18, 26]. The initial description of Arctic-like lineage of CDV dates back to the late 1980s, when epizootics were observed in seals in Northern Europe and Siberia [27–29]. The strains of the Arctic-like lineage are closely related to the CDV strains in North America, China and Greenland and were recently isolated from domestic dogs in Hungary [30] and domestic dogs and wolves in Southern Italy [31, 32]. The present study shows how fast these strains can spread to central European countries by import of infected domestic dogs.

The present study indicates that rescue dogs may also play an important role in the spread of vector-borne infections to central European countries. *Babesia spp., L. infantum* or *D. immitis* infections were detected in four of the 13 rescue dogs. Several dogs were reported to be free of these pathogens based on the laboratory certificates provided by the animal welfare organisation. In the case of Dog 13, *D. immitis* infection was diagnosed in this study using the antigen enzyme immunoassay and the Knott test in November 2013. The dog was found to be negative in the Knott test in August 2013. The *D. immitis* antigen and Knott tests are known to turn positive not before five to eight months after infection [33]; therefore, the infection could have been missed in the first testing. *L. infantum* serology specimen, which was positive in Dog 4, has similar limitations in that negative serological results cannot exclude infection and seroconversion can occur many months after infection [34]. Future dog owners should be properly informed concerning the limitations of these tests and the costs of treatment for vector-borne infections.

## Conclusion

The present study highlights the risks of spreading contagious viral and vector-borne infections by a non-selective import of sick and unvaccinated dogs or dogs with incomplete vaccination from Eastern European countries. The animal welfare organisations should be thoroughly informed concerning the critical importance of complete vaccination schedules and quarantine measures in animal shelters to combat the outbreaks and the spread of viral pathogens to other countries. Dog owners in Switzerland should be educated regarding the risk of and the potential costs of adopting sick dogs or dogs with incomplete vaccination and the critical importance of sufficiently high vaccination rates in domestic dogs in Switzerland.

## Methods

### Study design

The present study describes a distemper outbreak in fifteen dogs originating from an animal shelter in Kecskemét, Hungary, and the prospective follow-up of the infected animals. The dogs were imported to Switzerland by an animal welfare organisation on October 22, 2013. One dog had to be euthanised within a several days of arrival. No data on that dog were available. Of the remaining fourteen dogs, the signalment, vaccination and medical history and clinical signs were recorded (Table 1) and dogs were monitored for six months.

### Sample and data collection and sample processing

Within three weeks of arrival, a clinical examination was performed in 13 of the 14 dogs at the small animal clinics of the Vetsuisse Faculty, University of Zurich (Dogs 1 and 2) or Bern (Dog 3), by a private veterinarian (Dogs 4, 8 and 12) or by the first author (BW) during a visit of the dogs at their private homes (Dogs 5 to 7, 9 to 11 and 13, Table 1). No clinical examination or sample collection was possible in Dog 14 because of aggressive behaviour. In five dogs (Dogs 1 to 4 and 13), radiographic examinations of the thorax were performed at the small animal clinic of the Vetsuisse Faculty of Zurich (Dogs 1, 2 and 13), of the Vetsuisse Faculty of Bern (Dog 3), or by a private veterinarian (Dog 4). In Dog 13, echocardiography was performed at the division of cardiology, Vetsuisse Faculty, University of Zurich.

Nasal and/or conjunctival swabs were collected from 13 dogs for CDV-specific real-time RT-qPCR and rectal swabs from 12 dogs for CPV real-time qPCR as indicated in Tables 4 and 5. EDTA blood and serum samples were collected to obtain haematology and blood biochemistry results, and for CDV real-time RT-qPCR and testing for vector-borne infections (*Babesia* spp., *E. canis, L. infantum* and *D. immitis)* as indicated in Tables 2 through 5. All of the samples were processed within 12 h of collection. In 10 of the 11 CDV-infected dogs (Dogs 1 to 3 and 5 to 11), monthly follow-up examinations for CDV were performed from conjunctival, nasal and oropharyngeal swabs (Table 4). The swabs were collected by the owner based on written and image instructions regarding how to collect and ship the samples. All of the swabs were sent by priority mail to the Clinical Laboratory, Vetsuisse Faculty, University of Zurich, within one day of collection. Follow-up was continued in each dog until the dog tested CDV PCR-negative, except for Dog 10, in which the owner declined further testing despite a PCR-positive result at three months of follow-up.

### Haematology and blood biochemistry

Haematology and blood biochemistry tests were performed at the Clinical Laboratory, Vetsuisse Faculty, University of Zurich (Dogs 1 and 2, 5 to 11 and 13), at the Clinical Diagnostic Laboratory, Vetsuisse Faculty, University of Bern (Dog 3), at a private laboratory (Dog 4: Alomed, Radolfzell-Böhringen, Germany) or by a private veterinarian (Dog 8, Tables 2 and 3). The laboratory’s own device-specific reference intervals were applied; for dogs aged 6 to 8 months (Dogs 2, 5 to 7), published reference intervals for phosphorous, alkaline phosphatase, urea and creatinine were used [35].

### Total nucleic acid (TNA) extraction

At the time of the initial examinations, conjunctival, nasal or rectal swabs were incubated for 10 min in 300 μl of phosphate buffered saline (PBS) at 40 °C; the swabs were subsequently turned upside down and centrifuged for 1 min at 6440 × *g* and the supernatant was used for TNA extraction (see below). For the follow-up examinations, the two conjunctival and the two nasal swabs, respectively, were pooled in a total of 400 μl of PBS before incubation at 40 °C for 10 min and TNA extraction. The TNA extraction was performed from 100 μl of EDTA blood, 200 μl of swab supernatant or vaccine material that was resuspended in 500 μl of PBS (see below) using the MagNa Pure LC (Roche Diagnostics AG, Rotkreuz, Switzerland) and the MagNa Pure LC TNA Isolation Kit (Roche Diagnostics) following the manufacturer’s instructions. With each batch of extraction, a negative control consisting of 200 μl of PBS was used to monitor for cross-contamination. The TNA was stored at −80 °C until PCR analysis was performed.

### PCR assays and sequencing

For CDV testing, a published real-time RT-qPCR assay was used as previously described [36]. For the detection of CPV, a published real-time qPCR assay developed for the detection of feline parvovirus (FPV) was applied [37]. The assay amplifies a 107 bp sequence of the highly conserved VP1/VP2 gene region using the following primers and probe: PV3294f: 5′-ACTGCATCATTGATGGTTGCA-3′; PV3400r: 5′-GGTATGGTTGGTTTCCATGGA-3′ PV3375p: 5′-FAM-CCCAATGTCTCAGATCTCATAGCTGCTGG-6-TAMRA-3′. Sequence comparison revealed that the primer and probe-binding sites in the VP1/VP2 gene region of FPV and CPV showed > 99 % sequence identity (GenBank accession numbers M38246, M38245, M19296, M74849, M74852, M24000, M24003, KM457142, JQ268284). During CDV follow-up, a published canine (c)GAPDH PCR assay was applied to ensure that the quality of the swabs collected by the dog owners was adequate [38]. During follow-up, dogs were stated CDV-negative if they tested PCR-negative for CDV in conjunctival, nasal and oropharyngeal swabs and the swabs showed a cGAPDH threshold cycle below 32. All of the real-time PCR reactions were run using an ABI 7500Fast Real-Time PCR system (Applied Biosystems, Rotkreuz, Switzerland). Negative and positive controls were included in each PCR run.

To ensure that the CDV real-time RT-qPCR signal was due to infection and was not due to a recent vaccination, a published real-time RT-qPCR system based on the CDV M gene and M-F intergenic region was used [17]. The primers of the PCR assay amplify the MLV vaccine and wild type CDV strains, whereas the probe of the assay only binds to the MLV vaccine strains (Fig. 3). Because no sequence data have been published on the M gene and the M-F intergenic region of CDV U39 strain contained in the vaccine used in the rescue dogs (Biocan® DHPPi & L, Bioveta), the latter vaccine, together with two other CDV vaccines that are commercially available in Switzerland (Nobivac® DHHPi, MSD Animal Health; Canigen® SHA2PPi, Vibac) were tested to confirm that the CDV strains contained in these vaccines are detected by the assay. The TNA from the three vaccines and from the conjunctival or nasal swabs from 10 of 11 CDV PCR-positive dogs (Dogs 1 to 3 and 5 to 11) were subjected to real-time RT-qPCR. The PCR products were separated using 2.5 % agarose gel and appropriate-sized bands (294 bp) cut out, extracted using the MinElute® Gel extraction kit (Qiagen, Hombrechtikon, Switzerland) and sequenced (Microsynth, Balgach, Switzerland).

The complete haemagglutinin (HA) genes of the CDV isolates from five dogs were sequenced (Dogs 1, 5, 6, 9 and 10). The TNA extracted from the conjunctival swabs was used as a template. For amplification, five previously published primer pairs were used [39]; single nucleotides in one primer pair (472f and 1172r) had to be changed because of mismatches within the primer binding sites for the five CDV isolates (472f_new: 5'- CTGTACATCACCAAGTCATA-3' and 1172r_new: 5'-TAGAATACCATCTTGTGAAT-3'). Reverse transcription was performed using the High Capacity cDNA reverse transcription kit (Applied Biosystems) according to the manufacturer’s instructions. The PCR amplification was conducted using 5 μl of 5 x HF PCR buffer (Finnzymes, BioConcept, Allschwil, Switzerland), 500 nM each primer, 200 μM each dNTP (Sigma-Aldrich, Buchs, Switzerland), 1 U Phusion High-Fidelity DNA Polymerase (Finnzymes), and 2.5 μl template cDNA made up to 25 μl with water. The thermal cycling conditions comprised 98 °C for 2 min, 35 cycles at 98 °C for 30 s, 50 °C for 30 s, 65 °C for 1 min, and a final elongation at 72 °C for 3 min. After the PCR run, the amplification products were separated using 2 % agarose gel; appropriately sized products were excised and purified using the MinElute Gel Extraction Kit (Qiagen). Direct sequencing of the purified amplicons was performed using the amplification primers in a commercial laboratory (Microsynth, Balgach, Switzerland) under standard conditions.

### Testing for vector-borne infections

Vector-borne infections were tested in 13 dogs, as shown in Table 5. The analyses were performed at the Clinical Laboratory (for *E. canis*) and the Institute of Parasitology (for *Babesia spp., L. infantum* and *D. immitis*) of the Vetsuisse Faculty, University of Zurich, and at private laboratories (Dog 8: Labor am Zugersee, Hünenberg, Switzerland; Dog 4: Alomed; Dog 12: IDEXX Diavet AG, Bäch, Switzerland) (Table 5). The laboratories’ own reference values were used for defining the positive, negative and intermediate results. The microscopic blood smear evaluation for the presence of *Babesia spp.* organisms was conducted in Dog 3 by the private veterinarian and in Dog 4 by a commercial laboratory (Alomed) (Table 5).

### Sequence editing and phylogenetic analyses

The obtained sequences were edited and aligned with a consensus sequence using Geneious Version 7.1.8 [40]. Only the nucleotides available for all of the included sequences (2005 nucleotides of the HA gene) were used to calculate the percent nucleotide identities and perform the phylogenetic analyses. For the phylogenetic analyses, the sequences were aligned with known distemper sequences from GenBank (see Fig. 4) using Geneious Version 7.1.8. A bootstrap phylogenetic tree demonstrating the relationship between the isolates was created using the maximum-likelihood method [41] and a distance matrix corrected for nucleotide substitutions based on the Kimura 2-parameter model [42]. The dataset was resampled 1000 times to generate bootstrap values. The phylogenetic and molecular evolutionary analyses were conducted using the MEGA version 6 [43].

### Nucleotide sequence accession numbers

Nucleotide sequences obtained in this study have been submitted to GenBank under accession numbers KR002657 to KR002671.

## Abbreviations

CDV: Canine distemper virus
MLV: Modified live virus
ANIS: Animal identity service
CPV: Canine parvovirus
RT: Reverse transcription
q: Quantitative
TNA: Total nucleic acid
PBS: Phosphate buffered saline
FPV: Feline parvovirus
c: Canine
HA: Haemagglutinin
PDV: Phocine distemper virus
